# Improvement of gluten‐free bread and cake properties using natural hydrocolloids: A review

**DOI:** 10.1002/fsn3.1245

**Published:** 2019-10-17

**Authors:** Fakhreddin Salehi

**Affiliations:** ^1^ Department of Biosystems Engineering Faculty of Agriculture Bu‐Ali Sina University Hamedan Iran

**Keywords:** basil seed, celiac disease, gluten‐free, gums, wild sage seed, xanthan

## Abstract

The main wheat component responsible for bread and cake quality is gluten. Celiac disease is an autoimmune digestive disease that is caused by the digestion of gluten, and the only treatment of this disease is a gluten‐free diet. Various gluten‐free formulations (composite and wheatless flours) have applied gums (as gluten substitutes) to mimic the viscoelastic properties of gluten. In the bakery products, gums have been used to improve dough performance, bread and cake characteristics, textural and sensorial quality, and extension the products shelf life. This paper reviews the effect of the most common and new hydrocolloids (balangu seed, wild sage seed, basil seed, cress seed, xanthan, guar, starch carrageenan, methylcellulose, carboxy methyl cellulose, hydroxyl propyl methyl cellulose, and locust bean gums) on the rheological, physicochemical, textural, and quality characteristics of gluten‐free breads and cakes. Gums affect gelatinization and retrogradation of starch through a strong association of amylose with gum, resulting in a decrease in the retrogradation of starch. Gums addition increased volume and porosity of the breads and cakes and resulted in softer products.

## 1 INTRODUCTION

The main wheat component responsible for bread and cake quality is gluten. Gluten plays a principal role in bread and cake development by giving cohesiveness and promoting the retention of the CO_2_ produced during fermentation. The gas expansion causes wheat breads to gain volume and attain acceptable crumb texture (Elgeti, Jekle, & Becker, 2015; Le‐Bail et al., 2011; Martínez & Gomez, 2017).

Gluten replacement is nowadays one of the most challenging issues for food technology since a lifelong gluten‐free diet is essential for patients having gluten‐sensitive enteropathy or celiac disease. Celiac disease, a disorder induced by gluten intolerance, has prompted food scientists in the world to search for gluten‐free alternatives to wheat flour for breads and cakes making (De Simas et al., 2009; Gobbetti, Rizzello, Cagno, & Angelis, 2007; Sahraiyan, Naghipour, Karimi, & Ghiafe Davoodi, 2013). Gluten‐sensitive enteropathy is characterized by small intestinal malabsorption of nutrients after ingestion of prolamins from wheat and also other Triticum species like rye, barley, oat, and their crossbred varieties (Elgeti et al., 2015; Farrell & Kelly, 2001; Parra, Ribotta, & Ferrero, 2015). As diagnosis methods are improved, revealing the high incidence of gluten intolerance in the world, the demand for novel, nutritious, and high‐quality gluten‐free foods also ascends (Deora, Deswal, & Mishra, 2014; Gallagher, Gormley, & Arendt, 2004; Sahraiyan et al., 2013; Stojceska & Butler, 2012). McCarty, Gallagher, Gormley, Schober, and Arendt (2005) optimized gluten‐free bread formulation based on mainly rice flour, potato starch, and skim milk powder.

Some food additives such as gums should be added to the gluten‐free breads and cakes to obtain the desired quality. Gums are an important group of polysaccharides, generally ramified with high molecular weight. Water‐soluble gums also are known as “hydrocolloid” are used for various applications as dietary fiber, packaging films, texture modifiers, thickeners, gelling agents, stabilizers, emulsifiers, and coating agents (Salehi, 2019b; Salehi & Kashaninejad, 2014b; Salehi, Kashaninejad, & Behshad, 2014; Williams & Phillips, 2000). They are found in various sources (Table 1), and their use in baking is related to their capability to control rheology and the texture of aqueous suspensions (Juszczak et al., 2012; Salehi & Kashaninejad, 2015b; Williams & Phillips, 2000). Gums are added to the food products mainly for their thickening and gelling properties. In addition, they are used to improve mouth feel and to change the viscosity of solutions due to their high polymeric nature and the interactions between polymer chains when they are dissolved or dispersed (Anton & Artfield, 2008; Turabi, Sumnu, & Sahin, 2008; Yaseen, Herald, Aramouni, & Alavi, 2005).

**Table 1:** Main sources of natural gums (Salehi, 2019c; Williams & Phillips, 2000)

Botanical	Trees: Cellulose
	Tree gum exudates: Arabic gum, karaya gum, ghatti gum, tragacanth gum
	Plants: Starch, pectin, cellulose
	Seeds: Basil seed gum (*Ocimum basilicum*); balangu seed gum (*Lallemantia royleana*); wild sage seed gum (*Salvia macrosiphon*); cress seed gum (*Lepidium sativum*)
	Tubers: Konjac manan
Algal	Red seaweeds: Agar, carrageenan
	Brown seaweeds: Alginate
Microbial	Xanthan gum, curdlan, dextran, gellan gum, cellulose
Animal	Gelatin, caseinate, whey protein, chitosan

High‐quality breads and cakes have various attributes, including high volume, uniform crumb structure, tenderness, shelf life, and tolerance to staling. The quality of finished breads and cakes can be influenced by the addition of substances that affect these properties, as gums do. Several studies have been carried out showing the potential use of hydrocolloids in breads, biscuits, cakes, and pasta formulation (Anton & Artfield, 2008; Bojnanska, Smitalova, & Vollmannova, 2016; Gómez, Ronda, Caballero, Blanco, & Rosell, 2007; Salehi, 2017). The hydrocolloids improved the water holding capacity of the starch aqueous system. The increase in the moisture content in the eggless cake with gums is explained by the ability of hydrocolloids to hydrate at room temperature and its self‐interactions without competing with gluten proteins and starchy polysaccharides for the water available in the system (Ashwini, Jyotsna, & Indrani, 2009; Nawrocka, Mis, & Szymanska‐Chargot, 2016; Salehi, 2019c; Wang, Tao, Jin, & Xu, 2016).

Several studies have been carried out showing the potential use of gums in breads and cakes making including gluten‐free bread, wheat bread, whole wheat bread, rye bread, protein‐fortified starch bread, and frozen bread dough (Bourekoua et al., 2018; Gómez et al., 2007; Heinio et al., 2016; Ho & Noor Aziah, 2013; Pecivova, Dula, & Hrabe, 2013; Salehi, 2017). Majzoobi, Vosooghi Poor, Mesbahi, Jamalian, and Farahnaky (2017) studied the effects of carrot pomace powder and a mixture of pectin and xanthan on the quality of gluten‐free batter and cakes. Addition of different gums to bakery products significantly increased water absorption capacity (Kaur, Sandhu, Arora, & Sharma, 2015; Salehi, 2017). In another study, Pahwa, Kaur, and Puri (2016) studied the influence of hydrocolloids on the quality of major flat breads. Sharadanant and Khan (2003) reported that acacia gum increased the loaf volume and improved bread characteristics such as texture, cell wall structure, and softness. The physicochemical, sensory, and transport properties of foods are largely dependent on crumb structure. Crumb structure affects the appearance of crumb volume and texture of bakery product. Gums addition increased volume and porosity of the cakes and resulted in softer products. In addition, the bulk density of products increased slightly after incorporation of gums (Salehi, 2017; Turabi, Sumnu, & Sahin, 2010). Batters are complex mixtures of flour and various ingredients. The properties of batter systems are further altered by the addition of hydrocolloids (Kaur et al., 2015). According to Gomez, Ronda, Caballero, Blanco, and Rosell (2007), the influence of hydrocolloids on the final cake volume is due to increase in batter viscosity that slows down the rate of gas diffusion and allows its retention during the early stages of baking (Salehi & Kashaninejad, 2018). The use of hydrocolloids as antistaling agents in bread has also been studied (Davidou, Meste, Debever, & Bekaert, 1996; Turabi et al., 2008, 2010). Hager and Arendt (2013) investigated the influence of HPMC, xanthan gums, and their combination on loaf specific volume, crumb hardness, and crumb grain characteristics of gluten‐free breads based on rice, maize, teff, and buckwheat. Rice, which has a very low level of gluten, low levels of sodium, fat, protein, and fiber, and a high amount of easily digested carbohydrates, is one of the most frequently used cereals as a wheat substitute in gluten‐free food products (Kang, Choi, & Choi, 1997; Sivaramakrishnan, Senge, & Chattopadhyay, 2004; Sumnu, Koksel, Sahin, Basman, & Meda, 2009). Kang et al. (1997) reported that many gum types including hydroxyl propyl methyl cellulose (HPMC), locust bean gum, guar gum, carrageenan, xanthan gum, and agar resulted in acceptable rice (*Oryza sativa*) breads. Rheological properties of gluten‐free rice bread formulations were studied by Demirkesen, Mert, Sumnu, and Sahin (2010). The rice dough containing different gums at 25°C showed shear‐thinning behavior with a flow behavior index (n) ranging from 0.33 to 0.68 and consistency index (K) ranging from 2.75 to 61.7 Pa.s^n^.

Comparison of breads and cakes prepared from that of gluten‐free flour alone and those with gums added revealed that the incorporation of gum resulted in significant improvement in texture, volume, color, appearance, flavor, and overall acceptability. The present study summarized the effect of different gums on the rheological, textural, and physical properties and quality of various types of gluten‐free breads and cakes.

## 2 GUMS SOURCES

The natural gums are categorized based on their origins, chemical structures, and behaviors. Gums are known as complex polysaccharides from various sources, for example, endosperm of plant seeds (e.g., guar gum), plant exudates (e.g., tragacanth), tree exudates (e.g., gum arabic), sea weed extracts (e.g., agar), bacteria (e.g., xanthan gum), seed gums (e.g., basil seed gum), and animal sources (e.g., gelatin) (Table 1) (Galla & Dubasi, 2010; Salehi et al., 2014; Vinod, Sashidhar, Sarma, & Satyanarayana Raju, 2010; Williams & Phillips, 2000).

An interesting feature of several bacteria is the production of exopolysaccharides with a structure and function similar to that of hydrocolloids. Rühmkorf et al. (2012) demonstrated a positive effect of exopolysaccharides produced by four different strains of lactobacilli on the specific volume of gluten‐free bread. There are a large number of plant species that are being “cultivated” that are capable of producing gums which can be implemented in the food industry as additives. Various parts of the plant have surface cells containing gums, mucilage, and fiber and protein compounds. Some of the plant seeds have surface cells containing gums, mucilage, fiber, and protein. Plant gum exudates are produced by several plants as a result of the protection mechanisms against mechanical or microbial injury (Mirhosseini & Amid, 2012; Rana et al., 2011; Williams & Phillips, 2000).

## 3 STARCH

Starches and flours have extensive microstructural differences at granular structural scales, which can influence their capacity to generate gluten‐free breads with high‐quality standards. However, the use of scanning electron microscopy (*SEM*) as a tool to view gluten‐free doughs and breads has been reported on very few occasions. Composition and physical properties of the different flours or starches are shown in Table 2 (Liu et al., 2018; Martínez & Gomez, 2017).

**Table 2:** Composition and physical characteristics of the different flours and starches (Martínez & Gomez, 2017)

Starch‐based ingredient	Moisture (%)	Protein (%)	Water binding capacity (g water/g solid)	Pasting temperature (°C)	Peak viscosity (cp)
Maize flour	9.37	6.1	1.421	73.55	3,535
Rice flour	8.70	7.8	1.291	70.20	3,082
Maize starch	10.54	‐	1.337	75.20	4,988
Wheat starch	11.10	‐	0.626	57.40	5,697
Potato starch	14.66	‐	0.171	65.30	12,143

Mechanistic relations between the evolution of the starch–flour structure, dough rheology, and bread quality were studied in gluten‐free bread making by Martínez and Gomez (2017). Micrographs showed that the small wheat starch granules filled the spaces of the big granules, forming a uniform starch–hydrocolloid matrix. This granular advantage decreased the consistency and increased the uniformity of wheat‐starch‐based doughs throughout fermentation. The viscoelastic properties of the different doughs strongly influenced the bread volume, and the crumb texture of gluten‐free breads and starch‐based breads showed higher specific volume and lower hardness (Table 3).

**Table 3:** The effect of the origin of the starch‐based ingredient and the baking time on the volume and texture of gluten‐free breads (Liu et al., 2018; Martínez & Gomez, 2017)

Starch‐based ingredient	Specific volume (ml/g)	Hardness (N)	Elasticity	Cohesiveness	Resilience
Maize flour	2.18	6.733	0.750	0.322	0.141
Rice flour	4.69	0.732	0.833	0.576	0.327
Maize starch	7.14	1.250	0.955	0.560	0.415
Wheat starch	8.40	0.957	0.983	0.681	0.568
Potato starch	6.64	0.877	0.956	0.588	0.405

## 4 XANTHAN GUM

Some studies have reported the use of xanthan gum as gluten substitutes in the formulation of gluten‐free breads and cakes. Xanthan gum is completely soluble in both hot and cold water, and imparts high solution viscosity at low concentrations. Xanthan gum mixed with other gums is the most widely used in bakery products (Kaur et al., 2015; Kim & Yoo, 2006; Ozkoc & Seyhun, 2015). In the study of Miller and Hoseney (1993), it was shown that xanthan gum significantly improved the cake volume. Xanthan and carboxy methyl cellulose gums have been used as gluten substitutes in the formulation of gluten‐free breads due to their polymeric structure (Movahhed, Ranjbar, & Ahmadi Chenarbon, 2014). Özboy (2002) added five different commercial food‐grade hydrocolloids including carrageenan, xanthan–guar blend, xanthan–carrageenan blend, guar–carrageenan blend, and locust bean gum, to the corn starch to produce low phenylalanine starch–gum bread for phenylketonuria patients.

The functionality of gums of different origin and chemical structure (sodium alginate, carrageenan, pectin, hydroxyl propyl methyl cellulose, locust bean gum, guar gum, and xanthan gum) on yellow layer cake quality and their potential use in retarding the staling process have been studied by Gómez et al. (2007). Physical characteristics of fresh cakes and their evolution in time were notably influenced by the type of gum involved. In general, except when pectin was used, the overall acceptability of yellow layer cakes was always improved by hydrocolloid addition. Regarding shelf life, xanthan was able to maintain unaltered all texture parameters during storage.

Quality properties of buckwheat flour were compared with refined wheat flour, and gums (guar, acacia, xanthan, and tragacanth) were added in the concentration of 1 g/100 g to buckwheat by Kaur et al. (2015). Incorporation of gums to buckwheat significantly (*p* < .05) affected various quality properties like water absorption capacity, oil absorption capacity, and emulsion activity. The incorporation of gums to buckwheat improved the sensory scores. Biscuits prepared from buckwheat included with gums were observed to have higher moisture content, diameter, thickness, weight, and decreased fracture strength. Among the gums, the addition of xanthan gum resulted in significant improvement in biscuit color, appearance, flavor, and overall acceptability. Incorporation of gums to buckwheat flour significantly improved emulsion activity with xanthan gum addition resulting in a maximum increase. Quantitative analysis of macro‐ and microstructure of gluten‐free rice cakes containing different types of gums was studied by Turabi et al. (2010). The gum types used were xanthan, guar, locust bean, k‐carrageenan, and xanthan–guar blend. It was observed that both additions of different types of gums affected the pore area fraction and percent number of pores of the rice cakes. The highest pore area fraction was obtained in cakes containing xanthan and xanthan–guar blend.

The incidence of celiac disease, which is a gluten intolerance disease, has made food scientists search for gluten‐free flours instead of wheat flour for bread making. Xanthan gum has been added to composite cassava–wheat dough and bread to improve its viscoelastic properties (Shittu, Aminu, & Abulude, 2009). Higher gum content increased the oven spring, loaf volume, crumb softness, percent cell area, and overall sensory acceptability of composite cassava–wheat bread. 1% xanthan gum was found sufficient to retard moisture loss and firming of the bread crumb.

Rheological information is valuable in product development. Rheological properties and quality of rice cakes formulated with different gums and an emulsifier blend were evaluated by Turabi et al. (2008). Synergistic interaction between guar and xanthan gum resulted in a higher viscosity of cake batters as compared to other gums, and HPMC‐containing batters had the lowest apparent viscosity values. Emulsifier blend and gum interaction were found to be significant in affecting apparent viscosity. The highest specific volume values in the absence of emulsifier blend were obtained for cakes containing only xanthan gum. Rice starch–xanthan gum mixtures at 25°C have shown shear‐thinning flow behavior, and both consistency index and apparent viscosity of mixtures increased with the increase in gum concentration. Rheological characteristics of batters were modeled by Power law and Casson models, and both models were suitable to explain the rheological behavior of rice cakes. In another study, the Casson model was also found to be a suitable model to explain the rheological behavior of rice cake batters (Kim & Yoo, 2006). In the study of Chun and Yoo (2004), the rice flour dispersions showed a high shear‐thinning behavior (pseudoplastic) with low magnitudes of Casson yield stress. Rice starch–xanthan gum mixtures at 25°C have shown shear‐thinning flow behavior, and both consistency index and apparent viscosity of mixtures increased with the increase in gum concentration (Kim & Yoo, 2006).

In some works, an increase in the bread volume was appreciated as viscoelastic moduli (G' and G'') decreased (Mancebo, San Miguel, Martínez, & Gomez, 2015). However, studying different hydrocolloids, Mancebo et al. (2015) observed that the creep‐recovery technique could be more suitable than oscillatory shear tests to predict bread volume. Increasing concentration of xanthan gum resulted in softer crumb in freshly gluten‐free bread (Guarda, Rosell, Benedito, & Galotto, 2004).

Different gum combinations containing xanthan and pectin with amylase and sucrose have been used in order to improve dough and bread quality. The improved rheological characteristics can be achieved using complex formulations, which contain hydrocolloids combined with amylolytic enzymes, nonamylolytic enzymes, and emulsifiers. In addition, the negative effects of freezing and defrosting of dough can be reduced by incorporating hydrocolloids in dough (Akbarian et al., 2015).

In bakery products, dried fruit pomace can be added to replace flour, sugar, or fat and thus reduce energy load while enhancing fiber and antioxidant contents (Quiles, Campbell, Struck, Rohm, & Hernando, 2018; Salehi, 2019a, 2019d). Singh, Kaur, and Singh (2016) research was undertaken to explore application of black carrot pomace dietary fiber concentrate (at three levels of 3%, 6%, and 9%) and xanthan gum (0.5%) in gluten‐free rice muffins. Black carrot pomace showed higher water absorption and oil absorption capacities than rice flour. Incorporation of black carrot pomace increased total dietary fiber content and decreased the L* and b* values, water activity (aw), specific volume, and firmness. Muffins prepared with 6% black carrot pomace incorporation and xanthan gum were the most acceptable and can be used as viable functional ingredients in the preparation of gluten‐free muffins. The effects of different fiber sources (apple, orange, and carrot pomace powders) on gluten‐free batter rheology and quality characteristics of rice flour‐based cakes were studied by Kırbaş, Kumcuoglu, and Tavman (2019). Their results showed that the apparent viscosity, elastic modulus (G′), and viscous modulus (G″) of the cake batter increased with increasing pomace powders content. Also, the addition of pomace powder increased batter specific gravity and crumb hardness, and decreased the specific volume of cakes. The sensory properties of the cake samples were investigated concerning color, texture, appearance, flavor, and overall acceptability, and those with 5% orange pomace powder received the highest acceptance scores from the panelists.

## 5 GUAR GUM

Bread and cake volume and crumb texture and appearance are the most widely studied characteristics for assessing final product quality. Some natural gums such as guar and xanthan gums should be added to the bakery food products to obtain the desired quality (Ibañez & Ferrero, 2003; Mirhosseini & Amid, 2012; Sumnu et al., 2009). One of the plant gums is guar with 19%–43% gum (Ibañez & Ferrero, 2003; Mirhosseini & Amid, 2012). The ash content of guar gum (11.9%) was higher than that of arabic gum (1.2%) and xanthan gum (1.5%) (Cui & Mazza, 1996). Guar gum, a polysaccharide produced from the seed endosperm of *Cyamopsis tetragonolobus*, is highly viscous at low concentrations and has useful thickening, stabilizing, and water binding properties. It is also used to improve mixing tolerance, to prolong the shelf life of the end product through its moisture retention property and to prevent syneresis in frozen food products (Mandala, 2005; Sahraiyan et al., 2013; Thombare, Jha, Mishra, & Siddiqui, 2016). In the bakery products, guar gum has been used to improve mixing and extension of shelf life of the products through moisture retention and prevention of syneresis in frozen foods and pie fillings (Kaur et al., 2015). Yoo, Kim, and Yoo (2005) found that rice starch–galactomannan mixtures containing locust bean and guar gums showed high shear‐thinning flow behaviors with high Casson yield stress. In the study of Casas, Mohedano, and García‐Ochoa (2000), the mixtures of xanthan and guar gum showed a higher viscosity than that occurred in each gum individually. This synergistic effect is not always valid for all gum types. For example, xanthan–k‐carrageenan blend gave lower apparent viscosity. Guar gum has a higher molecular weight than locust bean gum; therefore, its solution showed a higher viscosity than the solution of locust bean gum (Casas et al., 2000). Turabi et al. (2008) reported that rice cakes containing xanthan–guar gum blend without emulsifier blend were the firmest due to the thickening of the crumb walls surrounding the air spaces. Gómez et al. (2007) showed that guar gum led to the hardest conventionally baked wheat cakes. To develop low‐calorie soft dough biscuits, fat in the biscuit formulation was reduced from 20% to 6% (Sudha, Srivastava, Vetrimani, & Leelavathi, 2007). Addition of glycerol monostearate and guar gum had a positive effect on dough consistency and hardness. Further improvement in biscuit texture was noted when guar gum was used with maltodextrin.

The effect of guar, arabic, carrageenan, xanthan, and HPMC gums on the quality properties of south Indian parotta was studied separately at the level of 0.5% by Smitha, Rajiv, Begum, and Indrani (2008). The amylograph peak viscosity increased with all the gums. Addition of gums increased farinograph water absorption. The extensograph resistance to extension at 135 min increased with the addition of hydrocolloids. Addition of xanthan, carrageenan, and guar gums decreased extensibility, while arabic and HPMC gums increased extensibility. Among the hydrocolloids tried, guar gum brought about the greatest improvement in the quality of parotta.

The effects of xanthan and guar gums on staling of gluten‐free rice cakes baked in different ovens were studied by Sumnu et al. (2009). Gums were added at concentrations of 0.3% and 1.0%. For the preparation of gum blend, 0.5% xanthan gum was mixed with 0.5% guar gum. To understand the staling behavior of gluten‐free rice cakes, they were stored at 22 ± 2°C for 120 hr. Xanthan–guar gum blend decreased hardness, weight loss, retrogradation enthalpy, and the change in setback viscosity values of cakes during storage for both types of ovens as compared to control formulation.

## 6 K‐CARRAGEENAN GUM

k‐carrageenan, which is extracted from red seaweeds, also gave low values of apparent viscosity, although it is used in bread making as texture improver (Rosell, Rojas, & Barber, 2001). k‐carrageenan's ability to reduce firmness and to increase the specific volume of baked products could not help maintaining the desired structure of gluten‐free rice cake (Turabi et al., 2008). The effect of k‐carrageenan, xanthan, and guar gums added in making frozen dough on the characteristics of bread making was studied by Lee, Lee, Lee, Cho, and Kim (2001). The bread with k‐carrageenan showed the highest sensory score during the frozen storage. Their results summarized that k‐carrageenan was most effective in the protection from the degradation of the quality of frozen dough during the frozen storage. Rojas, Rosell, and Barber (1999) also reported that k‐carrageenan, xanthan, and alginate decreased pasting temperature. Leon et al. (2000) studied the interactions between different isoforms of carrageenans and gluten proteins by IR spectroscopy and SDS‐PAGE, and reported that *Lambda carrageenan* (the most sulfated isoform) could interact better with gluten proteins due to its higher hydration capacity and its particular conformation.

## 7 METHYLCELLULOSE

Methylcellulose (MC) is extensively used in the batter industry. It is high molecular weight water‐soluble carbohydrate biopolymer with the ability to form gels and thickening in aqueous systems. MC is cellulose derivative hydrocolloids. MC can gel when heated, but return to its original viscosity when it is cooled (Sanz, Fernandez, Salvador, Munoz, & Fiszman, 2005). Effects of MC, xanthan gum, and carboxy methyl cellulose (CMC) on thermal properties of batter systems were studied by Xue and Ngadi (2009). The hydrocolloids shifted gelatinization temperature (*T*_G_) upwards, depressed glass transition temperature (*T*_g_), and increased melting peak temperature (*T*_m_) of batters. The effect of these hydrocolloids on glass transition temperature was more pronounced in raw samples (freezing–cooking process) than in cooked samples and increased with increasing levels of CMC and MC used in the formulations. Batters with MC showed increased total melting enthalpies (∆*H*_m_) for all the thermal processes.

## 8 CARBOXY METHYL CELLULOSE GUM

Natural hydrocolloids improve mixing and extension of shelf life of the bakery products through moisture retention and prevention of syneresis in frozen foods and pie fillings (Ozkoc & Seyhun, 2015). Carboxy methyl cellulose (CMC) forms a three‐dimensional network with an ability to link water molecules within the system. They form and provide a barrier coating during heating leading to a reduction in water loss and oil uptake (Andrew, 2004). Cato, Rafael, Gan, and Small (2002) found that fine white and ground rice flours gave gluten‐free breads of good quality when used in combination with CMC (0.8%) and HPMC (3.3%).

With regard to gluten‐free breads, Lazaridou, Duta, Papageorgiou, Belc, and Biliaderis (2007) investigated the effect of gums (pectin, CMC, agarose, xanthan, and oat β‐glucan) on dough rheology and bread quality parameters. The formulation was based on rice flour, corn starch, and sodium caseinate. It was shown that xanthan had the most pronounced effect on viscoelastic properties yielding strengthened doughs; addition of xanthan to the gluten‐free formulation resulted in a farinograph curve typical of wheat flour doughs. In addition, among the preparations supplemented with hydrocolloids the elasticity and resistance to deformation of dough, as determined by oscillatory and creep measurements, followed the order of xanthan > CMC > pectin > agarose > β‐glucan. In another study, the effect of xanthan and CMC on rheological properties of gluten‐free bread dough was studied by Sadeghnia, Azizi, Seyedain Ardebili, and Mohammadi (2016). Increasing the level of xanthan led to increasing water absorption significantly in comparison with CMC. Xanthan and CMC gums decreased and increased dough time development, respectively. Addition of xanthan resulted in a farinogram which resembled that of a standard farinogram obtained by wheat flour. In dough dynamic measuring, hydrocolloids caused higher viscoelastic modulus and increasing the level of them made them greater. CMC and guar gum have been added to rye bread recipes to improve the quality of the bread (Mettler & Seibel, 1993). Doughs with prebiotics have been assessed by Angioloni and Collar (2008). This high‐fiber (up to 12%) dough included different hydrocolloids blended or not with prebiotic oligosaccharides. When 10% wheat flour was replaced with CMC and prebiotic oligosaccharides, singly and in hydrocolloid/oligosaccharide binary blends (70:30 *w*/*w*), a more regular dough structure was observed.

## 9 HYDROXYL PROPYL METHYL CELLULOSE GUM

Gums are added to the food products mainly for their thickening and gelling properties. Also, they are used to improve mouth feel and to change the viscosity of solutions due to their high polymeric nature and the interactions between polymer chains when they are dissolved or dispersed (Turabi et al., 2008). Sivaramakrishnan et al. (2004) found that the rice dough containing hydroxyl propyl methyl cellulose (HPMC) had similar rheological properties as that of wheat flour dough and was suitable for making bread. Rheological information is valuable in product development. Cake batter must retain sufficient viscosity to prevent the incorporated air bubbles from rising to the surface (Lu, Lee, Mau, & Lin, 2010). In the study of Rosell et al. (2001), HPMC and k‐carrageenan reduced the crumb firmness for control bread. According to Bell (1990), HPMC forms interfacial films at the boundaries of the gas cells that confer some stability to the cells against the gas expansion and processing condition changes. HPMC has also been used as an improver in wheat bread, yielding higher specific volume, softer crumb, and enhanced sensory characteristics (Rosell et al., 2001).

Modified celluloses HPMC and CMC (0.1%–0.5% flour basis) have been widely reported to be effective in decreasing hardening rate and final crumb firmness values (Ferrero, 2017). Armero and Collar (1998) found that the effect of HPMC and CMC depended on the type of flour and bread‐making process. For white breads made by the sourdough method, the CMC effect was greater at short storage times, and that of HPMC was more effective at longer ones. HPMC was also effective as crumb softener in the straight dough process. By differential scanning calorimetry (DSC) assays, Barcenas and Rosell (2005) reported a lower retrogradation index for samples containing HPMC (0.5% flour basis) stored at 4°C. Guarda et al. (2004) investigated the effect of different gums on fresh gluten‐free bread quality as well as their effects on bread staling. It was found that the effect of the different types of gums varied and a concentration of 0.1% was enough to cause the observed effects. HPMC made all‐round improvement on all the bread properties studied. While all the gums were also able to reduce the dehydration rate of bread crumb during storage, alginate and HPMC showed exceptional retardation of staling.

## 10 LOCUST BEAN GUM

Good quality bakery products can thus be prepared from wheat flour with the incorporation of gums. Gums are added to minimize nondesired changes in crumb texture during storage. Turabi et al. (2008) studied the rheological properties and quality of rice cakes formulated with different gums and an emulsifier blend. Their results showed that when locust bean gum was used in the bakery products formulations, higher apparent viscosity values were obtained (Turabi et al., 2008). Angioloni and Collar (2009) demonstrated the ability of locust bean gum in combination with prebiotic fibers fructo‐oligosaccharides or gluco‐oligosaccharides (at 6%–12% level of replacement), to delay crumb firming during storage. Sharadanant and Khan (2003) found that the addition of locust bean gum (1%–3% flour basis) to a frozen dough led to lower proof times, higher resistance to extension, and higher specific bread volumes with softer crumbs than the control without gum.

## 11 BALANGU SEED GUM

In recent years, the demand for plant‐based gums in food systems, medicines, and drug delivery systems has been considerably increased because they are the most notable ingredient in liquid and semisolid foods (Salehi & Kashaninejad, 2015b). The plant gum exudates and seed gums are the complex polysaccharides/carbohydrate polymers commonly used as a dietary fiber, texture modifiers, gelling and thickening agent, foaming and coating agent, packaging film, emulsifier, stabilizer, and drug delivery agent. Balangu seed (*Lallemantia royleana*) is a mucilaginous endemic plant which is grown in different regions of Asia, Europe, and the Middle East, especially in various regions of Iran. The extracted balangu seed gum has a high molecular weight and rather flexible chain. There has been some research into the effect of balangu seed gum on the properties of bread, ice cream, desserts, and emulsions (Mirhosseini & Amid, 2012; Sahraiyan et al., 2014; Salehi & Kashaninejad, 2014a; Salehi et al., 2014). Salehi, Amin Ekhlas, Pavee, and Zandi (2018) investigated the effect of balangu seed gum on rheological, physical, and sensory properties of gluten‐free rice cake. With increasing the Balangu seed gum from 0% to 1.5%, rice cake batter viscosity at a shear rate of 10 s^−1^ ware increased from 13.16 to 22.32 Pa.s. The moisture content and volume of cakes were increased with increasing gum percentage. According to the sensory evaluation results, samples containing 1.5% Balangu seed gum had the highest total acceptance score. With increasing Balangu gum, brightness of cakes increased due to increasing volume, as well as decreased yellowing of the samples. The L*, a*, and b* indexes for a sample containing 1.5% gum were 85.01, −1.37, and 37.56, respectively. In another study, Sahraiyan et al. (2014) studied the effect of balangu seed gum on quantitative and qualitative of sorghum gluten‐free bread. Their results showed that the moisture content and L* value were increased by adding this gum. The highest specific volume and overall acceptance in the sensory evaluation were observed in the sample containing 0.5% balangu seed gum. In addition, the lowest firmness was observed in this sample.

## 12 WILD SAGE SEED GUM

The genus *Salvia* (*Labiatae*) is a mucilaginous endemic plant and contains more than 700 species. Wild sage (*Salvia macrosiphon*) seeds are round small seeds, with a mucilage layer which could swell in water, giving viscous suspension properties which are comparable with commercial food hydrocolloids (Salehi & Kashaninejad, 2014b). Some potential of the wild sage seed gum as a new source of hydrocolloid has been recently investigated by Salehi and Kashaninejad (2015a).

Shelke, Faubion, and Hoseney (1990) reported that lower viscosity of the batter during heating is one of the reasons for decreased end product volume. It is possible that, in the presence of a less viscous batter, carbon dioxide evolved and water vapor produced might not be trapped in the air cells during baking, thus resulting in the cakes with low volume. Salehi (2017) determined the rheological, physical, and sensory properties of apple cake formulated with four different levels of wild sage seed gum (0%, 0.5%, 1.0%, and 1.5%). With increasing gum levels significantly increased the volume of cake, while the density values were decreased (*p* < .05). Apple cake batters formulated with wild sage seed gum showed pseudoplastic and thixotropic behavior. The apparent viscosity of cake batter significantly (*p* < .05) increased with increasing gum levels. The crumb color of apple cake was affected by the addition of gum. The apple cake with 1.5% gum exhibited a color, with L*, a*, and b* equal to 85.36, 1.53, and 35.32, respectively. 1.0% wild sage seed gum was suggested to use in apple cake formulation.

## 13 BASIL SEED GUM

Seeds hydrocolloids have good functional attributes such as viscosity, foaming, emulsifying, gelling, solubility, and textural improvement (Salehi, 2017; Zameni, Kashaninejad, Aalami, & Salehi, 2015). Basil seed (*Ocimum basilicum L.*) has reasonable content of gum with outstanding functional properties which are comparable with some other commercial food hydrocolloids (Zameni et al., 2015). Basil seed gum is a polysaccharide extracted from basil seed by using either cold water extraction (Salehi & Kashaninejad, 2017; Salehi, Kashaninejad, Tadayyon, & Arabameri, 2015; Zameni et al., 2015). The effect of basil seed gum (at four levels 0%, 0.5%, 1%, and 1.5%) on rheological, physical, and sensorial properties of rice cake was studied by Salehi, Gohari Ardabili, Satorabi, and Souri (2017). Trained panelists evaluated the sensory quality of the cake using a nine‐point hedonic scale. With increasing the basil seed gum from 0% to 1.5%, rice cake batter viscosity at a shear rate of 20 s^−1^ ware increased from 9.82 to 49.6 Pa.s. The moisture content and volume of cakes were increased with increasing gum percentage (Table 4). With increasing basil gum, brightness of cakes increased due to increasing volume, in addition, decreased yellowing of the samples. The L*, a*, and b* indexes for a sample containing 1.5% gum were 88.35, −1.71, and 37.89, respectively. In terms of sensorial scores, the quality of gluten‐free rice cakes prepared by incorporating 1% basil seed gum to rice flour was found best.

**Table 4:** Physical characteristics of rice sponge cakes with different concentration of Basil seed gum (Salehi et al., 2017)

Gum concentration (%)	Ash	Volume (cm^3^)	Density (kg/m^3^)	Moisture (%)	Weight after baking (g)
0	0.400 ± 0.01^a^	67.35 ± 0.53^c^	362 ± 4.21^a^	16.1 ± 0.26^c^	24.39 ± 0.13^b^
0.5	0.390 ± 0.01^a^	68.50 ± 0.77^c^	358 ± 3.82^b^	16.8 ± 0.55^bc^	24.54 ± 0.11^b^
1	0.403 ± 0.01^a^	70.39 ± 0.34^b^	364 ± 4.72^a^	17.5 ± 0.31^b^	25.62 ± 0.08^a^
1.5	0.393 ± 0.01^a^	72.73 ± 0.61^a^	355 ± 2.64^b^	18.2 ± 0.32^a^	25.83 ± 0.06^a^

Means with different letter within same columns are significantly different (*p* < .05).

## 14 CRESS SEED GUM

*Lepidium sativum* (cress) is an annual herb, which belongs to the Cruciferae family, which grows widely in the Middle East, Europe, and USA. *Lepidium sativum* seed (cress seed) exhibits quick water adsorption when soaked in water and produces a large amount of mucilaginous substance, and a gum of high molecular weight has been identified (Sahraiyan et al., 2013). Studies connecting gluten‐free dough rheology with the quality of the resultant bread are scarce. The effects of cress seed and guar gums (at 0%, 0.3%, 0.6%, and 1% *w*/*w*) in rice–wheat flour were studied by Sahraiyan et al. (2013). The evaluation of dough rheology showed that water absorption, dough development time, dough stability, and viscosity all increased with the addition of hydrocolloids alone or in a combination. The mixing tolerance index and gelatinization temperature decreased with an increased hydrocolloid concentration. Firmness decreased with increasing hydrocolloid concentration and increased with increasing storage time. The sensory evaluation by a consumer panel gave a higher score for overall acceptability to 0.3% guar and 0.3% cress seed gums sample.

## 15 CONCLUSION

Breads and cakes are traditional food generally prepared from wheat flour. The main wheat components responsible for bread and cake quality are gluten and are avoided in the diet of celiac disease patients. Recently, the market of gluten‐free breads and cakes has expanded (use of composite and wheat‐free flours) and substantial efforts are underway to enhance their quality. For the production of gluten‐free breads and cakes, the absence of gluten is critical and challenging in regard to the bread structure. Various gluten‐free formulations have applied gums to mimic the viscoelastic properties of gluten. Gums comprise several water‐soluble polysaccharides with varied chemical structures providing a range of functional properties that make them suitable for this application. They modify the pasting properties of starch. Also, gums can improve food taste, mouth feel, and texture, retard starch retrogradation, improve moisture retention, and enhance the overall quality of the gluten‐free breads and cakes during storage. Positive effects of xanthan, HPMC, carrageenan, alginate, guar, and seeds gums have been reported, at levels varying from 0.25% to 2.0% flour basis (Table 5).

**Table 5:** The best percent of gums for using in the bakery products

Selected gums	Product type	Used percent of gums (flour basis)	Suggested percent of gum (flour basis)	References
Balangu seed	Rice cake	0%, 0.5%, 1.0% and 1.5%	1.5% balangu seed	Salehi et al., 2018
Balangu seed gum	Gluten‐free bread	0%, 0.25%, 0.5%, 0.75% and 1.0%	0.5 balangu seed gum	Sahraiyan et al., 2014
Guar, acacia, xanthan, and tragacanth	Gluten‐free biscuits	1%	1% xanthan	Kaur et al., 2015
Xanthan, guar, locust bean, k‐carrageenan, and xanthan–guar	Rice cakes	1%	1% xanthan or 1% xanthan–guar blend	Turabi et al., 2010
Xanthan, guar, locust bean, k‐carrageenan, HPMC, xanthan–guar, and xanthan–k‐carrageenan	Rice cakes	1%	1% xanthan	Turabi et al., 2008
Cress seed and guar	Composite rice–wheat bread	0%, 0.3%, 0.6% and 1%	0.3% guar and 0.3% cress seed	Sahraiyan et al., 2013
Xanthan gum	Cassava–wheat dough and bread	0%, 1% and 2%	1% xanthan	Shittu et al., 2009
Alginate and HPMC	Gluten‐free bread	0.1%	0.1% HPMC	Guarda et al., 2004
Pectin, CMC, agarose, xanthan, and oat β‐glucan	Gluten‐free bread (corn and rice flour)	1.0% and 2.0%	1.0 xanthan	Lazaridou et al., 2007
Basil seed	Rice cake	0%, 0.5%, 1.0% and 1.5%	1% basil seed	Salehi et al., 2017
Xanthan and CMC	Gluten‐free bread	0.5%, 1.0% and 1.5%	1.5% xanthan	Sadeghnia et al., 2016

## CONFLICT OF INTEREST

The authors declare that they do not have any conflict of interest.

## ETHICAL APPROVAL

This study does not involve any human or animal testing.

## INFORMED CONSENT

Written informed consent was obtained from all study participants.

## References

[fsn31245-bib-0001] Akbarian, M. , Dehkordi, M. S. M. , Ghasemkhani, N. , Koladoozi, M. , Niknam, O. , & Morshedi, A. (2015). Hydrocolloids and Cryoprotectant used in Frozen Dough and Effect of Freezing on Yeast Survival and Dough Structure: A Review. International Journal of Life Sciences, 9(3), 1–7. 10.3126/ijls.v9i3.12439

[fsn31245-bib-0002] Andrew, C. H. (2004). Hydrocolloids: Practical guides for the food industry. St. Paul, MN: Eagan Press Handbook.

[fsn31245-bib-0003] Angioloni, A. , & Collar, C. (2008). Functional response of diluted dough matrixes in high‐fibre systems: A viscometric and rheological approach. Food Research International, 41, 803–812. 10.1016/j.foodres.2008.07.003

[fsn31245-bib-0004] Angioloni, A. , & Collar, C. (2009). Gel, dough and fibre enriched fresh breads: Relationships between quality features and staling kinetics. Journal of Food Engineering, 91, 526–532. 10.1016/j.jfoodeng.2008.09.033

[fsn31245-bib-0005] Anton, A. A. , & Artfield, S. D. (2008). Hydrocolloids in gluten‐free breads: A review. International Journal of Food Sciences and Nutrition, 59(1), 11–23. 10.1080/09637480701625630 18097842

[fsn31245-bib-0006] Armero, E. , & Collar, C. (1998). Crumb firming kinetics of wheat breads with antistaling additives. Journal of Cereal Science, 28, 165–174. 10.1006/jcrs.1998.0190

[fsn31245-bib-0007] Ashwini, A. , Jyotsna, R. , & Indrani, D. (2009). Effect of hydrocolloids and emulsifiers on the rheological, microstructural and quality characteristics of eggless cake. Food Hydrocolloids, 23, 700–707. 10.1016/j.foodhyd.2008.06.002

[fsn31245-bib-0008] Barcenas, M. E. , & Rosell, C. M. (2005). Effect of HPMC addition on the microstructure, quality and aging of wheat bread. Food Hydrocolloids, 19, 1037–1043. 10.1016/j.foodhyd.2005.01.005

[fsn31245-bib-0009] Bell, D. A. (1990). Methylcellulose as structure enhancer in bread baking. Cereal Foods World, 35, 1001–1006.

[fsn31245-bib-0010] Bojnanska, T. , Smitalova, J. , & Vollmannova, A. (2016). Effect of the addition of hydrocolloids on the rheological and baking properties of the products with added spelt flour (*Triticum spelta l.*). Potravinarstvo, 10, 157–163.

[fsn31245-bib-0011] Bourekoua, H. , Różyło, R. , Benatallah, L. , Wójtowicz, A. , Łysiak, G. , Zidoune, M. N. , & Sujak, A. (2018). Characteristics of gluten‐free bread: Quality improvement by the addition of starches/hydrocolloids and their combinations using a definitive screening design. European Food Research and Technology, 244(2), 345–354. 10.1007/s00217-017-2960-9

[fsn31245-bib-0012] Casas, J. A. , Mohedano, A. F. , & García‐Ochoa, F. (2000). Viscosity of guar gum and xanthan/guar gum mixture solutions. Journal of the Science of Food and Agriculture, 80(12), 1722–1727. 10.1002/1097-0010(20000915)80:12<1722:AID-JSFA708>3.0.CO;2-X

[fsn31245-bib-0013] Cato, L. , Rafael, L. , Gan, J. , & Small, D. (2002). The use of rice flour and hydrocolloid gums for gluten free breads. (pp. 304–308).Proceedings of the 51st Australian cereal chemistry conference

[fsn31245-bib-0014] Chun, S. , & Yoo, B. (2004). Rheological behavior of cooked rice flour dispersions in steady and dynamic shear. Journal of Food Engineering, 65(3), 363–370. 10.1016/j.jfoodeng.2004.01.035

[fsn31245-bib-0015] Cui, W. , & Mazza, G. (1996). Physicochemical characteristics of flaxseed gum. Food Research International, 29(3), 397–402. 10.1016/0963-9969(96)00005-1

[fsn31245-bib-0016] Davidou, S. , Le Meste, M. , Debever, E. , & Bekaert, D. (1996). A contribution to the study of staling of white bread: Effect of water and hydrocolloid. Food Hydrocolloids, 10(4), 375–383. 10.1016/S0268-005X(96)80016-6

[fsn31245-bib-0017] De Simas, K. N. , Vieira, L. D. N. , Podesta, R. , Müller, C. M. O. , Vieira, M. A. , & Beber, R. C. (2009). Effect of king palm (Archontophoenix alexandrae) flour incorporation on physicochemical and textural characteristics of gluten‐free cookies. International Journal of Food Science & Technology, 44, 531–538.

[fsn31245-bib-0018] Demirkesen, I. , Mert, B. , Sumnu, G. , & Sahin, S. (2010). Rheological properties of gluten‐free bread formulations. Journal of Food Engineering, 96(2), 295–303. 10.1016/j.jfoodeng.2009.08.004

[fsn31245-bib-0019] Deora, N. S. , Deswal, A. , & Mishra, H. N. (2014). Alternative approaches towards glutenfree dough development: Recent trends. Food Engineering Review, 6, 89–104. 10.1007/s12393-014-9079-6

[fsn31245-bib-0020] Elgeti, D. , Jekle, M. , & Becker, T. (2015). Strategies for the aeration of gluten‐free bread ‐A review. Trends in Food Science & Technology, 46, 75–84. 10.1016/j.tifs.2015.07.010

[fsn31245-bib-0021] Farrell, R. J. , & Kelly, C. P. (2001). Celiac sprue. The American Journal of Gastroenterology, 96(12), 3237–3246. 11774931 10.1111/j.1572-0241.2001.05320.x

[fsn31245-bib-0022] Ferrero, C. (2017). Hydrocolloids in wheat breadmaking: A concise review. Food Hydrocolloids, 68, 15–22. 10.1016/j.foodhyd.2016.11.044

[fsn31245-bib-0023] Galla, N. R. , & Dubasi, G. R. (2010). Chemical and functional characterization of gum karaya (*Sterculia urens L.*) seed meal. Food Hydrocolloids, 24, 479–485. 10.1016/j.foodhyd.2009.12.003

[fsn31245-bib-0024] Gallagher, E. , Gormley, T. R. , & Arendt, E. K. (2004). Recent advances in the formulation of gluten‐free cereal‐based products. Trends in Food Science & Technology, 15(3), 143–152. 10.1016/j.tifs.2003.09.012

[fsn31245-bib-0025] Gobbetti, M. , Rizzello, C. G. , Di Cagno, R. , & De Angelis, M. (2007). Sourdough lactobacilli and celiac disease. Food Microbiology, 24, 187–196. 10.1016/j.fm.2006.07.014 17008163

[fsn31245-bib-0026] Gomez, M. , Ronda, F. , Caballero, P. A. , Blanco, C. A. , & Rosell, C. M. (2007). Functionality of different hydrocolloids on the quality and shelf‐life of yellow layer cakes. Food Hydrocolloids, 21(2), 167–173. 10.1016/j.foodhyd.2006.03.012

[fsn31245-bib-0027] Gómez, M. , Ronda, F. , Caballero, P. A. , Blanco, C. A. , & Rosell, C. M. (2007). Functionality of different hydrocolloids on the quality and shelf‐life of yellow layer cakes. Food Hydrocolloids, 21(2), 167–173. 10.1016/j.foodhyd.2006.03.012

[fsn31245-bib-0028] Guarda, A. , Rosell, C. M. , Benedito, C. , & Galotto, M. J. (2004). Different hydrocolloids as bread improvers and antistaling agents. Food Hydrocolloids, 18, 241–247. 10.1016/S0268-005X(03)00080-8

[fsn31245-bib-0029] Hager, A. S. , & Arendt, E. K. (2013). Influence of hydroxypropylmethylcellulose (HPMC), xanthan gum and their combination on loaf specific volume, crumb hardness and crumb grain characteristics of gluten‐free breads based on rice, maize, teff and buckwheat. Food Hydrocolloids, 32, 195–203. 10.1016/j.foodhyd.2012.12.021

[fsn31245-bib-0030] Heinio, R. L. , Noort, M. W. J. , Katina, K. , Alam, S. A. , Sozer, N. , & de Kock, H. L. (2016). Sensory characteristics of wholegrain and bran‐rich cereal foods ‐ a review. Trends in Food Science and Technology, 47, 25–38.

[fsn31245-bib-0031] Ho, L. H. , & Noor Aziah, A. A. (2013). Dough mixing and thermal properties including the pasting profiles of composite flour blends with added hydrocolloids. International Food Research Journal, 20, 911–917.

[fsn31245-bib-0032] Ibañez, M. C. , & Ferrero, C. (2003). Extraction and characterization of the hydrocolloid from Prosopis flexuosa DC seeds. Food Research International, 36(5), 455–460. 10.1016/S0963-9969(02)00192-8

[fsn31245-bib-0033] Juszczak, L. , Witczak, T. , Ziobro, R. , Korus, J. , Cieślik, E. , & Witczak, M. (2012). Effect of inulin on rheological and thermal properties of gluten‐free dough. Carbohydrate Polymers, 90, 353–360. 10.1016/j.carbpol.2012.04.071 24751052

[fsn31245-bib-0034] Kang, M.‐Y. , Choi, Y.‐H. , & Choi, H.‐C. (1997). Effects of gums, fats and glutens adding on processing and quality of milled rice bread. Korean Journal of Food Science and Technology, 29(4), 700–704.

[fsn31245-bib-0035] Kaur, M. , Sandhu, K. S. , Arora, A. , & Sharma, A. . (2015). Gluten free biscuits prepared from buckwheat flour by incorporation of various gums: Physicochemical and sensory properties. LWT ‐ Food Science and Technology, 62(1), 628–632. 10.1016/j.lwt.2014.02.039.

[fsn31245-bib-0036] Kim, C. , & Yoo, B. (2006). Rheological properties of rice starch–xanthan gum mixtures. Journal of Food Engineering, 75(1), 120–128. 10.1016/j.jfoodeng.2005.04.002

[fsn31245-bib-0037] Kırbaş, Z. , Kumcuoglu, S. , & Tavman, S. (2019). Effects of apple, orange and carrot pomace powders on gluten‐free batter rheology and cake properties. Journal of Food Science and Technology, 56(2), 914–926. 10.1007/s13197-018-03554-z 30906049 PMC6400728

[fsn31245-bib-0038] Lazaridou, A. , Duta, D. , Papageorgiou, M. , Belc, N. , & Biliaderis, C. (2007). Effects of hydrocolloids on dough rheology and bread quality parameters in gluten‐free formulations. Journal of Food Engineering, 79(3), 1033–1047. 10.1016/j.jfoodeng.2006.03.032

[fsn31245-bib-0039] Le‐Bail, A. , Dessev, T. , Leray, D. , Lucas, T. , Mariani, S. , Mottollese, G. , & Jury, V. (2011). Influence of the amount of steaming during baking on the kinetic of heating and on selected quality attributes of bread. Journal of Food Engineering, 105, 379–385. 10.1016/j.jfoodeng.2011.03.001

[fsn31245-bib-0040] Lee, J.‐M. , Lee, M.‐K. , Lee, S.‐K. , Cho, N.‐J. , & Kim, S.‐M. (2001). Effect of Gums Added in Making Frozen Dough on the Characteristics of Bread‐making. Korean Journal of Food Science and Technology, 33(2), 190–194.

[fsn31245-bib-0041] Leon, A. E. , Ribotta, P. D. , Ausar, S. F. , Fernandez, C. , Landa, C. A. , & Beltramo, D. M. (2000). Interactions of different carrageenan isoforms and flour components in breadmaking. Journal of Agricultural and Food Chemistry, 48, 2634–2638. 10.1021/jf991340a 11032476

[fsn31245-bib-0042] Liu, X. , Mu, T. , Sun, H. , Zhang, M. , Chen, J. , & Fauconnier, M. L. (2018). Influence of different hydrocolloids on dough thermo‐mechanical properties and in vitro starch digestibility of gluten‐free steamed bread based on potato flour. Food Chemistry, 239, 1064–1074. 10.1016/j.foodchem.2017.07.047 28873523

[fsn31245-bib-0043] Lu, T.‐M. , Lee, C.‐C. , Mau, J.‐L. , & Lin, S.‐D. (2010). Quality and antioxidant property of green tea sponge cake. Food Chemistry, 119(3), 1090–1095. 10.1016/j.foodchem.2009.08.015

[fsn31245-bib-0044] Majzoobi, M. , Vosooghi Poor, Z. , Mesbahi, G. , Jamalian, J. , & Farahnaky, A. (2017). Effects of carrot pomace powder and a mixture of pectin and xanthan on the quality of gluten‐free batter and cakes. Journal of Texture Studies, 48(6), 616–623. 10.1111/jtxs.12276 28543050

[fsn31245-bib-0045] Mancebo, C. M. , San Miguel, M. A. , Martínez, M. M. , & Gomez, M. (2015). Optimisation of rheological properties of gluten‐free doughs with HPMC, psyllium and different levels of water. Journal of Cereal Science, 61, 8–15. 10.1016/j.jcs.2014.10.005

[fsn31245-bib-0046] Mandala, I. G. (2005). Physical properties of fresh and frozen stored, microwave reheated breads, containing hydrocolloids. Journal of Food Engineering, 66, 291–300. 10.1016/j.jfoodeng.2004.03.020

[fsn31245-bib-0047] Martínez, M. M. , & Gomez, M. (2017). Rheological and microstructural evolution of the most common gluten‐free flours and starches during bread fermentation and baking. Journal of Food Engineering, 197, 78–86. 10.1016/j.jfoodeng.2016.11.008

[fsn31245-bib-0048] McCarty, D. F. , Gallagher, E. , Gormley, T. R. , Schober, T. J. , & Arendt, E. K. (2005). Application of response surface methodology in the development of gluten‐free bread. Cereal Chemistry, 82(5), 609–615. 10.1094/CC-82-0609

[fsn31245-bib-0049] Mettler, E. , & Seibel, W. (1993). Effects of emulsifiers and hydrocolloids on whole wheat bread quality: A response surface methodology study. Cereal Chemistry, 70(3), 373–376.

[fsn31245-bib-0050] Miller, R. A. , & Hoseney, R. (1993). The role of xanthan gum in white layer cakes. Cereal chemistry (USA).

[fsn31245-bib-0051] Mirhosseini, H. , & Amid, B. T. (2012). A review study on chemical composition and molecular structure of newly plant gum exudates and seed gums. Food Research International, 46, 387–398. 10.1016/j.foodres.2011.11.017

[fsn31245-bib-0052] Movahhed, S. , Ranjbar, S. , & Ahmadi Chenarbon, H. (2014). Evaluation of chemical, staling and organoleptic properties of free – gluten cakes containing Xanthan and Carboxy Methyl Cellulose gums. Iranian Journal of Biosystems Engineering, 44(2), 173–178.

[fsn31245-bib-0053] Nawrocka, A. , Mis, A. , & Szymanska‐Chargot, M. (2016). Characteristics of relationships between structure of gluten proteins and dough rheology e influence of dietary fibres studied by FT‐Raman spectroscopy. Food Biophysics, 11, 81–90.

[fsn31245-bib-0054] Özboy, Ö. (2002). Development of corn starch‐gum bread for phenylketonuria patients. Food/Nahrung, 46(2), 87–91. 10.1002/1521-3803(20020301)46:2<87:AID-FOOD87>3.0.CO;2-Y 12017998

[fsn31245-bib-0055] Ozkoc, S. O. , & Seyhun, N. (2015). Effect of gum type and flaxseed concentration on quality of gluten‐free breads made from frozen dough baked in infrared‐microwave combination oven. Food and Bioprocess Technology, 8(12), 2500–2506. 10.1007/s11947-015-1615-8

[fsn31245-bib-0056] Pahwa, A. , Kaur, A. , & Puri, R. (2016). Influence of hydrocolloids on the quality of major flat breads: A review. Journal of Food Processing, 1(1), 1–9. 10.1155/2016/8750258

[fsn31245-bib-0057] Parra, A. F. R. , Ribotta, P. D. , & Ferrero, C. (2015). Apple pomace in gluten‐free formulations: Effect on rheology and product quality. International Journal of Food Science and Technology, 50(3), 682–690. 10.1111/ijfs.12662

[fsn31245-bib-0058] Pecivova, P. , Dula, P. , & Hrabe, J. (2013). The influence of pectin from apple and gum arabic from acacia tree on the quality of pizza. International Journal of Food Properties, 16, 1417–1428. 10.1080/10942912.2011.587931

[fsn31245-bib-0059] Quiles, A. , Campbell, G. M. , Struck, S. , Rohm, H. , & Hernando, I. (2018). Fiber from fruit pomace: A review of applications in cereal‐based products. Food Reviews International, 34(2), 162–181. 10.1080/87559129.2016.1261299

[fsn31245-bib-0060] Rana, V. , Rai, P. , Tiwary, A. K. , Singh, R. S. , Kennedy, J. F. , & Knill, C. J. (2011). Modified gums: Approaches and applications in drug delivery. Carbohydrate Polymers, 83, 1031–1047. 10.1016/j.carbpol.2010.09.010

[fsn31245-bib-0061] Rojas, J. , Rosell, C. , & De Barber, C. B. (1999). Pasting properties of different wheat flour‐hydrocolloid systems. Food Hydrocolloids, 13(1), 27–33. 10.1016/S0268-005X(98)00066-6

[fsn31245-bib-0062] Rosell, C. , Rojas, J. , & De Barber, C. B. (2001). Influence of hydrocolloids on dough rheology and bread quality. Food Hydrocolloids, 15(1), 75–81. 10.1016/S0268-005X(00)00054-0

[fsn31245-bib-0063] Rühmkorf, C. , Rübsam, H. , Becker, T. , Bork, C. , Voiges, K. , Mischnick, P. , … Vogel, R. F. (2012). Effect of structurally different microbial homoexopolysaccharides on the quality of gluten‐free bread. European Food Research and Technology, 235, 139–146. 10.1007/s00217-012-1746-3

[fsn31245-bib-0064] Sadeghnia, N. , Azizi, M. H. , Seyedain Ardebili, M. , & Mohammadi, M. (2016). Effect of xanthan and CMC on rheological properties of gluten free bread dough. Iranian Journal of Food Science and Technology, 13(51), 137–148.

[fsn31245-bib-0065] Sahraiyan, B. , Karimi, M. , Habibi Najafi, M. , Hadad Khodaparast, M. , Ghiafeh Davoodi, M. , Sheikholeslami, Z. , & Naghipour, F. (2014). The effect of Balangu Shirazi (Lallemantiaroyleana) gum on quantitative and qualitative of surghum gluten free bread. Iranian Journal of Food Science Technology. 42, 129–139.

[fsn31245-bib-0066] Sahraiyan, B. , Naghipour, F. , Karimi, M. , & Ghiafe Davoodi, M. (2013). Evaluation of Lepidium sativum seed and guar gum to improve dough rheology and quality parameters in composite riceewheat bread. Food Hydrocolloids, 30, 698–703.

[fsn31245-bib-0067] Salehi, F. (2017). Rheological and physical properties and quality of the new formulation of apple cake with wild sage seed gum (*Salvia macrosiphon*). Journal of Food Measurement and Characterization, 11(4), 2006–2012. 10.1007/s11694-017-9583-5

[fsn31245-bib-0068] Salehi, F. (2019a). Characterization of different mushrooms powder and its application in bakery products: A review. International Journal of Food Properties, 22(1), 1375–1385. 10.1080/10942912.2019.1650765

[fsn31245-bib-0069] Salehi, F. (2019b). Characterization of new biodegradable edible films and coatings based on seeds gum: A review. Journal of Packaging Technology and Research, 1(1), 1–10. 10.1007/s41783-019-00061-0

[fsn31245-bib-0070] Salehi, F. (2019c). Effect of common and new gums on the quality, physical and textural properties of bakery products: A review. Journal of Texture Studies, 1–12. 10.1111/jtxs.12482 31523824

[fsn31245-bib-0071] Salehi, F. (2019d). Recent applications and potential of infrared dryer systems for drying various agricultural products: A review. International Journal of Fruit Science, 1–17. 10.1080/15538362.2019.1616243

[fsn31245-bib-0072] Salehi, F. , Amin Ekhlas, S. , Pavee, S. , & Zandi, F. . (2018). Effect of balangu seed gum on rheological, physical and sensory properties of gluten free rice cake. Food Sciences and Nutrition, 15(4), 61-68.

[fsn31245-bib-0073] Salehi, F. , Gohari Ardabili, A. , Satorabi, M. , & Souri, F. (2017). Effect of basil seed gum on batter and rice cake properties. Iranian Journal of Food Science and Technology, 70(14), 315–323.

[fsn31245-bib-0074] Salehi, F. , & Kashaninejad, M. (2014a). Effect of different drying methods on rheological and textural properties of balangu seed gum. Drying Technology, 32(6), 720–727. 10.1080/07373937.2013.858264

[fsn31245-bib-0075] Salehi, F. , & Kashaninejad, M. (2014b). Kinetics and thermodynamics of gum extraction from wild sage seed. International Journal of Food Engineering, 10(4), 625–632. 10.1515/ijfe-2014-0079

[fsn31245-bib-0076] Salehi, F. , & Kashaninejad, M. (2015a). Effect of drying methods on rheological and textural properties, and color changes of wild sage seed gum. Journal of Food Science and Technology, 52(11), 7361–7368. 10.1007/s13197-015-1849-5 PMC4554639 26345008

[fsn31245-bib-0077] Salehi, F. , & Kashaninejad, M. (2015b). Static rheological study of ocimum basilicum seed gum. International Journal of Food Engineering, 11(1), 97–103. 10.1515/ijfe-2014-0189

[fsn31245-bib-0078] Salehi, F. , & Kashaninejad, M. (2017). Effect of drying methods on textural and rheological properties of basil seed gum. International Food Research Journal, 24(5), 2090–2096.

[fsn31245-bib-0079] Salehi, F. , & Kashaninejad, M. (2018). Modeling of xanthan gum effect on textural properties of carrot cake. Iranian Journal of Food Science and Technology, 75(15), 73–82.

[fsn31245-bib-0080] Salehi, F. , Kashaninejad, M. , & Behshad, V. (2014). Effect of sugars and salts on rheological properties of Balangu seed (Lallemantia royleana) gum. International Journal of Biological Macromolecules, 67, 16–21. 10.1016/j.ijbiomac.2014.03.001 24631549

[fsn31245-bib-0081] Salehi, F. , Kashaninejad, M. , Tadayyon, A. , & Arabameri, F. (2015). Modeling of extraction process of crude polysaccharides from Basil seeds (Ocimum basilicum l.) as affected by process variables. Journal of Food Science and Technology, 52(8), 5220–5227. 26243945 10.1007/s13197-014-1614-1 PMC4519519

[fsn31245-bib-0082] Sanz, T. , Fernandez, M. A. , Salvador, A. , Munoz, J. , & Fiszman, S. M. (2005). Thermogelation properties of methylcellulose (MC) and their effect on a batter formula. Food Hydrocolloids, 19, 141–147. 10.1016/j.foodhyd.2004.04.023

[fsn31245-bib-0083] Sharadanant, R. , & Khan, K. (2003). Effect of hydrophilic gums on the quality of frozen dough: II. Bread Characteristics. Cereal Chemistry, 80(6), 773–780. 10.1094/CCHEM.2003.80.6.773

[fsn31245-bib-0084] Shelke, K. , Faubion, J. A. , & Hoseney, R. C. (1990). The dynamics of cake making as studied by a combination of viscometry and electrical resistance oven heating. Cereal Chemistry, 67, 575–580.

[fsn31245-bib-0085] Shittu, T. A. , Aminu, R. A. , & Abulude, E. O. (2009). Functional effects of xanthan gum on composite cassavaewheat dough and bread. Food Hydrocolloids, 23, 2254–2260.

[fsn31245-bib-0086] Singh, J. P. , Kaur, A. , & Singh, N. (2016). Development of eggless gluten‐free rice muffins utilizing black carrot dietary fibre concentrate and xanthan gum. Journal of Food Science and Technology, 53(2), 1269–1278. 10.1007/s13197-015-2103-x 27162407 PMC4837742

[fsn31245-bib-0087] Sivaramakrishnan, H. P. , Senge, B. , & Chattopadhyay, P. (2004). Rheological properties of rice dough for making rice bread. Journal of Food Engineering, 62(1), 37–45. 10.1016/S0260-8774(03)00169-9

[fsn31245-bib-0088] Smitha, S. , Rajiv, J. , Begum, K. , & Indrani, D. (2008). Effect of hydrocolloids on rheological, microstructural and quality characteristics of parotta–an unleavened indian flat bread. Journal of Texture Studies, 39(3), 267–283. 10.1111/j.1745-4603.2008.00142.x

[fsn31245-bib-0089] Stojceska, V. , & Butler, F. (2012). Investigation of reported correlation coefficients between rheological properties of the wheat bread doughs and baking performance of the corresponding wheat flours. Trends in Food Science and Technology, 24, 13–18. 10.1016/j.tifs.2011.09.005

[fsn31245-bib-0090] Sudha, M. L. , Srivastava, A. K. , Vetrimani, R. , & Leelavathi, K. (2007). Fat replacement in soft dough biscuits: Its implications on dough rheology and biscuit quality. Journal of Food Engineering, 80, 922–930. 10.1016/j.jfoodeng.2006.08.006

[fsn31245-bib-0091] Sumnu, G. , Koksel, F. , Sahin, S. , Basman, A. , & Meda, V. (2009). The effects of xanthan and guar gums on staling of gluten‐free rice cakes baked in different ovens. International Journal of Food Science and Technology, 45(1), 87–93. 10.1111/j.1365-2621.2009.02107.x

[fsn31245-bib-0092] Thombare, N. , Jha, U. , Mishra, S. , & Siddiqui, M. Z. (2016). Guar gum as a promising starting material for diverse applications: A review. International Journal of Biological Macromolecules, 88, 361–372. 10.1016/j.ijbiomac.2016.04.001 27044346

[fsn31245-bib-0093] Turabi, E. , Sumnu, G. , & Sahin, S. (2008). Rheological properties and quality of rice cakes formulated with different gums and an emulsifier blend. Food Hydrocolloids, 22(2), 305–312. 10.1016/j.foodhyd.2006.11.016

[fsn31245-bib-0094] Turabi, E. , Sumnu, G. , & Sahin, S. (2010). Quantitative analysis of macro and micro‐structure of gluten‐free rice cakes containing different types of gums baked in different ovens. Food Hydrocolloids, 24(8), 755–762. 10.1016/j.foodhyd.2010.04.001

[fsn31245-bib-0095] Vinod, V. T. P. , Sashidhar, R. B. , Sarma, V. U. M. , & Satyanarayana Raju, S. (2010). Comparative amino acid and fatty acid compositions of edible gums kondagogu (*Cochlospermum gossypium*) and karaya (*Sterculia urens*). Food Chemistry, 123, 57–62. 10.1016/j.foodchem.2010.03.127

[fsn31245-bib-0096] Wang, P. , Tao, H. , Jin, Z. , & Xu, X. (2016). Impact of water extractable arabinoxylan from rye bran on the frozen steamed bread dough quality. Food Chemistry, 200, 117–124. 10.1016/j.foodchem.2016.01.027 26830568

[fsn31245-bib-0097] Williams, P. A. , & Phillips, G. O. (2000). Introduction to food hydrocolloids In Phillips G. O., & Williams P. A. (Eds.), Handbook of hydrocolloids. New York, NY: CRC Press.

[fsn31245-bib-0098] Xue, J. , & Ngadi, M. (2009). Effects of methylcellulose, xanthan gum and carboxymethylcellulose on thermal properties of batter systems formulated with different flour combinations. Food Hydrocolloids, 23, 286–295. 10.1016/j.foodhyd.2008.01.002

[fsn31245-bib-0099] Yaseen, E. I. , Herald, T. J. , Aramouni, F. M. , & Alavi, S. (2005). Rheological properties of selected gum solutions. Food Research International, 38(2), 111–119. 10.1016/j.foodres.2004.01.013

[fsn31245-bib-0100] Yoo, D. , Kim, C. , & Yoo, B. (2005). Steady and Dynamic Shear Rheology of Rice Starch‐Galactomannan Mixtures. Starch‐Stärke, 57(7), 310–318. 10.1002/star.200400390

[fsn31245-bib-0101] Zameni, A. , Kashaninejad, M. , Aalami, M. , & Salehi, F. (2015). Effect of thermal and freezing treatments on rheological, textural and color properties of basil seed gum. Journal of Food Science and Technology, 52(9), 5914–5921. 10.1007/s13197-014-1679-x 26345008 PMC4554639

